# The Principal Components of Adult Female Insole Shape Align Closely with Two of Its Classic Indicators

**DOI:** 10.1371/journal.pone.0133303

**Published:** 2015-08-26

**Authors:** Fred L. Bookstein, Jacqueline Domjanic

**Affiliations:** 1 Department of Anthropology, University of Vienna, Vienna, Austria; 2 Department of Clothing Technology, University of Zagreb, Zagreb, Croatia; Monash University, AUSTRALIA

## Abstract

The plantar surface of the human foot transmits the weight and dynamic force of the owner’s lower limbs to the ground and the reaction forces back to the musculoskeletal system. Its anatomical variation is intensely studied in such fields as sports medicine and orthopedic dysmorphology. Yet, strangely, the shape of the insole that accommodates this surface and elastically buffers these forces is neither an aspect of the conventional anthropometrics of feet nor an informative label on the packet that markets supplementary insoles. In this paper we pursue an earlier suggestion that insole form in vertical view be quantified in terms of the shape of the foot not at the plane of support (the “footprint”) but some two millimeters above that level. Using such sections extracted from laser scans of 158 feet of adult women from the University of Zagreb, in conjunction with an appropriate modification of today’s standard geometric morphometrics (GMM), we find that the sectioned form can be described by its size together with two meaningful relative warps of shape. The pattern of this shape variation is not novel. It is closely aligned with two of the standard footprint measurements, the Chippaux-Šmiřák arch index and the Clarke arch angle, whose geometrical foci (the former in the ball of the foot, the latter in the arch) it apparently combines. Thus a strong contemporary analysis complements but does not supplant the simpler anthropometric analyses of half a century ago, with implications for applied anthropology.

## Introduction

The foot of *Homo sapiens* must repeatedly absorb reaction forces during locomotion. In the normal foot, shod or unshod, much of this reaction is managed by the mechanics and the structural strength of the longitudinal arch. [1] A century ago, orthopedists, anthropologists and sports scientists began to investigate the configuration of this arch in order to prevent a variety of frequently encountered foot deformations and pathologies, such as the fallen arches that kept many conscripts out of World War I.

After that war, interest in physical education rose, and in its role in maintaining standards of general public health and well-being, especially in America. As Stafford [2] remarked, “The desire to be healthy [now] became fashionable.” Much of this new attention concentrated on the foot, sometimes to an absurd extent: thus Morton [3] claimed that more than 75% of American youth have had some sort of foot disorder. Stafford, basing his argument on the prevalence of flat feet, went on to emphasize the need for preventive and corrective physical education among civilized people. Only a very few reach adulthood with comfortable, efficient feet, he reported, owing to improper shoes, bodily weakness, or injuries to the lower limb. Not only evolutionary biology was to blame, but also civilization. Walking on hard floors and pavement forced the populace to use shoes and stockings, but the consequent restriction of normal foot action resulted in deformation and discomfort for millions. Stafford classified feet into three groups (normal, weak, and deformed), characterized each type, and proposed treatments.

This decade also saw the advent of the pedograph, a device originally intended for the use of shoe salesmen as an aid in fitting shoes and now used in sports science. [4] Much of the subsequent course of pedometry over the rest of the twentieth century pursued the problem of information transfer from this two-dimensional image, which is in reality a controlled footprint, to the three-dimensional representation at the core of the earlier concern with arch height (see the premature claim of Clarke [5], for instance, that the footprint, if taken properly, shows the height of the longitudinal arch). Schwartz [6] seems to have been the first to anticipate the measures persisting into the present paper when he introduced the footprint angle, characterized pretentiously as the chief mathematical measure of foot conditions available from footprints. His conjecture was that as arches are strengthened, the arch angle increases steadily, and so could be used as a proxy for therapeutic improvement over the course of various treatment regimens. Rogers [7] pursued such investigations further, but neither actually managed to provide a strict protocol for the placement of the two lines making up the angle that was to be measured. Clarke was honest about this, noting, “the footprint measure would become a more useful tool for physical educators if this line of best fit was definitely [sic] defined.”

Clarke tested this then-novel metrology on some students from Syracuse (New York, USA) University and claimed to have proven sufficient reliability and validity of the method for it to be considered a valuable aid in educational programs, medical treatment, and posture training. He was partially correct, in that a smaller angle is correlated with a lower arch. [8, 9] This plantographic method surely was more convenient than radiography and more reliable than palpation, and it supplied numbers, which could be converted into statistics, which generally impressed colleagues, clients, and customers. [10–13]

Continuing with this Whig history, we find the next forward step to be Chippaux’s, who, working in the French anthropological tradition, pursued additional characterizations of arch shape and its pathologies. [14] The arch angle was augmented by an additional parameter, the ratio of minimum to maximum width of the apparent arch outline measured perpendicular to the anteroposterior tangent of the footprint along its lateral edge. For a particularly good early example of a research application relying on this index, a 1960 study of Czech children and adolescents, see [15]. Later Šmahel [10] applied the same method to a sample of Czech adults. The combination of the Chippaux arch ratio and the Clarke arch angle provided a plausible basis for the classification of their sample into categories of arch types along with an ascertainment of their population frequencies. Of course the investigator must be concerned to control the weight borne by the foot whose footprint is being quantified. Where Šmahel required that the full body weight be borne by the foot under inspection, as in active striding, later investigators [11, 16–19, 9] would typically impose a condition of static bipedal loading instead. We have done the same.

The main clinical uses of the images that result from arch pedography and pedometry are to further a pair of quasimedical diagnoses (“*pes planus*,” “*pes cavus*”) and to pursue the implications for the design of appropriate arch inserts where necessary (see, e.g., [11, 20]). This therapeutic prosthesis, the insole, has a long history, back to the very first types of footwear (which often used layers of wool to cushion the feet). The early 20th-century adoption of factory-made shoes with different left-right lasts was associated with a wider exploration of the orthotic devices that could be inserted in shoes. Orthotics promised to correct various foot problems, improve foot function during locomotion, and relieve pain. Recent studies continue to address the issue of plantar pressure relief on sensitive feet [21–23] that can explain the increased popularity of insoles worldwide, along with the rapid advances in scanning and engineering technology and in materials science pertinent to design and fabrication of customized or custom-made insoles. [24, 25] However, the time and labor required for production of these objects make them more expensive than the mass-produced (prefabricated) insoles that fulfill needs of odor and moisture control or additional cushioning as well as (however approximately) the originally intended function of added arch support. [26] There is still market competition between personalized and prefabricated foot orthotics. A crucial step in rationalizing this competition would be an improved understanding of the shape variation of the arch outline supplied by pedography.

We present a retrospective view of this near-century of methodological development, beginning with a reanalysis of previously published data [1] demonstrating how the new morphometrics (geometric morphometrics, GMM) may be applied to a two-dimensional representation of a clinically important three-dimensional structure, the plantar surface of the foot. That earlier publication did not pursue comparisons of its findings with those of earlier methodologies. Our present follow-up computations fill the lacuna in that earlier investigation; but our conclusion is an unexpected one. Our main finding is indeed closely aligned with the gifted guesses of Clarke and Chippaux more than half a century ago, in that the best contemporary morphometric techniques of pattern analysis, applied to a good contemporary representation of the pedogram, yield two factors that can be directly interpreted as the sum and difference of those two early twentieth-century indices. In other words, although our computations closely parallel those of the earlier paper, our conclusion is actually the opposite: the new methods do not supplant, but rather complement, the insights of the founders of the anthropometry of the footprint.

## Materials and Methods

### Data

Data for this analysis combine information from sections of three-dimensional surface representations of 158 feet with additional linear and angular measurements and with responses to questionnaires.

The study was conducted among a sample of convenience comprising 83 female University of Zagreb (Croatia) students between 19 and 36 years of age scanned during January and February 2012 on the Pedus scanner in the Department of Clothing Technology at the University of Zagreb. To control for time-of-day effects, scanning was restricted to the hours between 11:00 and 16:00. Before scanning, each subject filled out a brief questionnaire informing us of her body weight and body height, shoe size, and frequency of wearing high heels, and also about any use of orthotics. (The restriction of the sample to females owed to the original research context of the project, an investigation of the consequences of wearing high heels for forefoot pain and long-term functional damage that might require orthotics for its amelioration.)

Morphometric data were acquired as detailed surface scans of each foot using a device specialized for that purpose (Pedus^*TM*^, Human Solutions, Germany). Each foot was scanned separately. During each of the two scanning intervals of about ten seconds, subjects stood upright with their weight borne symmetrically on both feet. Each scan produced a three-dimensional point cloud of some 60,000 points that was exported for analysis. In the Amira^*TM*^ software platform that is common to many anthropometry labs nowadays, the point cloud was converted to the form of a surface triangulation (.stl format), and then left and right footprints were produced as surface cuts two millimeters proximal to the bearing surface of the Pedus device. The value of 2 millimeters was chosen subjectively as a compromise between representation of the silhouette and sensitivity of the actual lower margin of the footprint to details of the stiffness of the skin in that region. (For more discussion of the interplay between two-dimensional and three-dimensional analyses in pedal surface data, see [27]. In the present context, of course, the comparison scalars to which we are referring, arch index and arch angle, are themselves only two-dimensional.) Left feet were reflected to the right side for all further analyses. For four of our 83 participants, at least one of their footprints could be classified by eye as either *pes cavus* or *pes planus*; the other 79 pairs made up the 158 feet of the present study.

There resulted six closed outlines per foot, one for each of the five toes and one for the remaining area of the footprint. Discarding the toes, we used Jim Rohlf’s program TPSdig2.0 (http://life.bio.sunysb.edu/morph/) to sample each of the 158 outlines of the main footprint at 36 approximately equally spaced sampling points, and resampled these 36-gons by sliding points along edge chords to the positions of minimum bending energy according to the algorithm of Bookstein [28]. The resulting locations are known in the GMM literature as *semilandmarks* or *slipped shape coordinates*. These 158 polygons were our primary morphometric resource.

### Arch measurements

**Fig 1:**
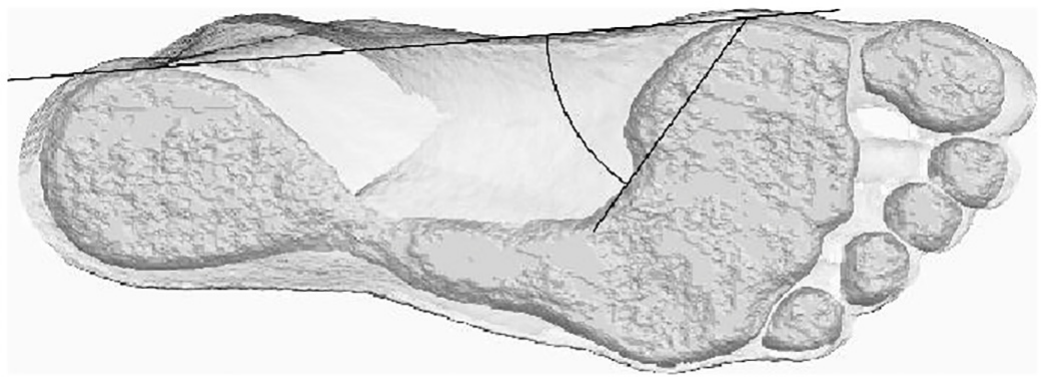
Operationalization of the arch angle, after the examples in Clarke [5]. The image, from Amira, shows a footprint and the associated section at 2 mm above the plane of support.

**Fig 2:**
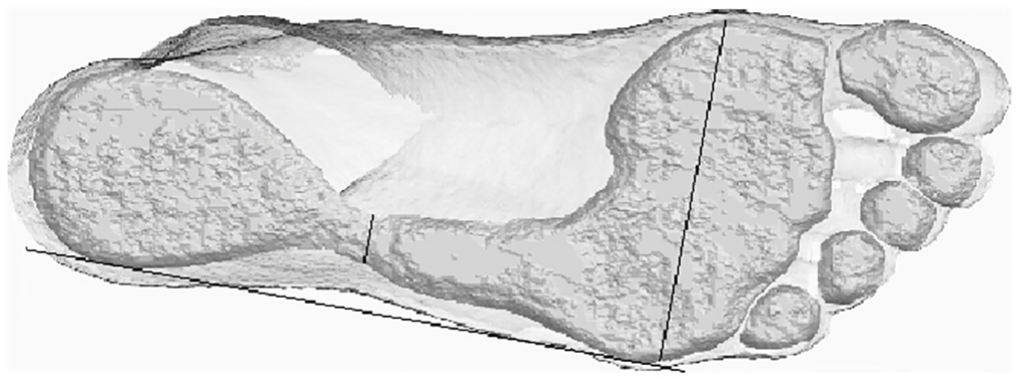
Amira operationalization of the arch ratio on the same image, implementing the approach of Šmiřák [15]. The quantity of interest is the ratio of the shorter to the longer transect shown in the figure. The anterior transect (Šmiřák’s A, denominator of the ratio) is constrained to be perpendicular to the lateral anteroposterior tangent to the specific surface section chosen here; it is not a function of the outer margin of the image (the silhouette of the foot). The posterior transect, Šmiřák’s B, is parallel to A.

In addition to the 36-point curves, we used Amira to calculate versions of the literature’s two standard parameters, the Clarke’s angle and the Chippaux-Šmiřák ratio. The Clarke’s angle we used is the one sometimes called “alpha” in the literature [12, 9] and sometimes just “the arch angle.” [29–31] It is the angle between the medial tangent to the footprint and the segment connecting the point of contact of the medial tangent in the anterior part of the footprint with the deepest point of the medial contour of the footprint outline. Our version of the Chippaux-Šmiřák Index was the one described by Šmahel [10]. See Figs 1 and 2.

### Statistical analyses

**Fig 3:**
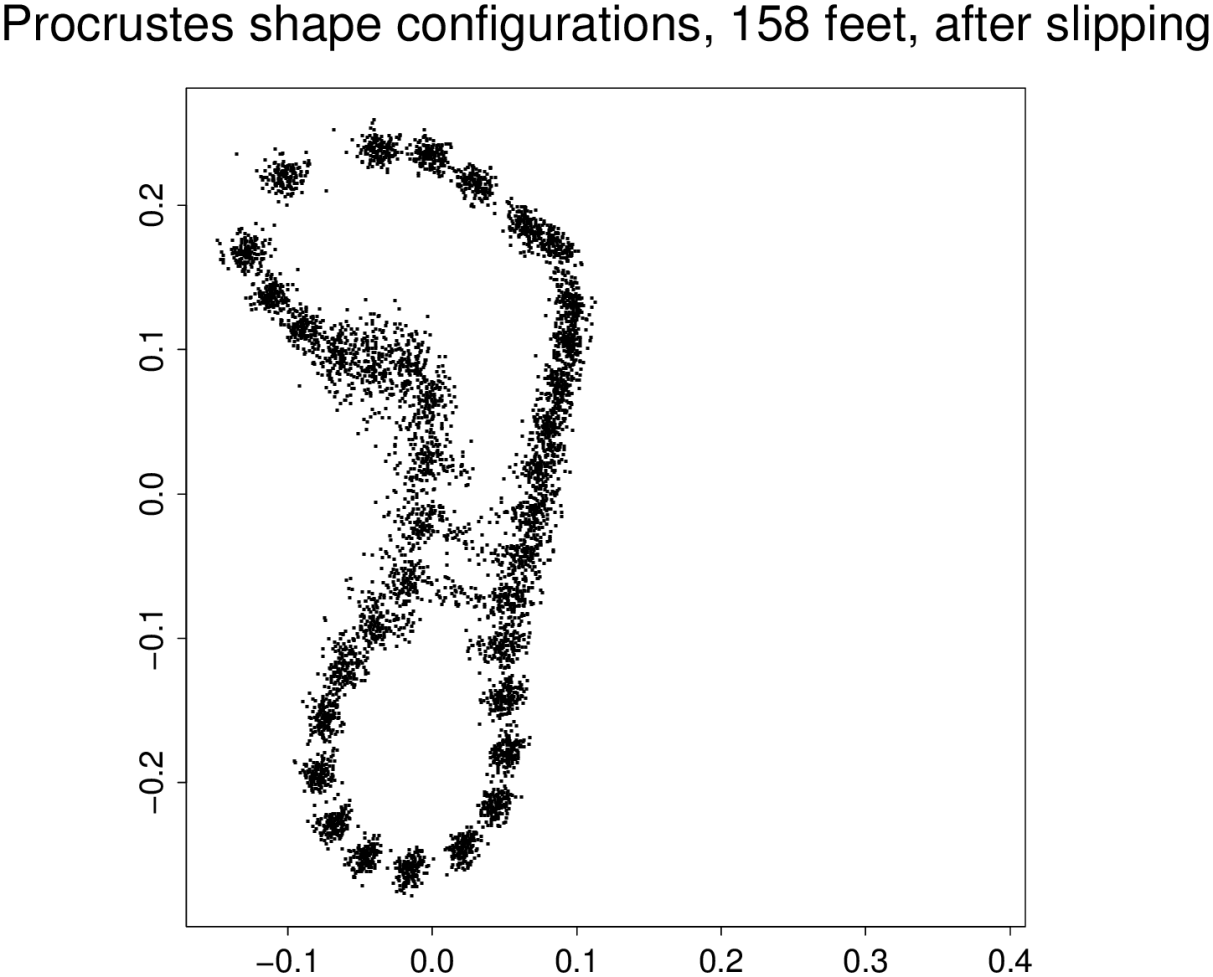
The data for the GMM computations. Shown are slipped Procrustes coordinates of 158 36-gons for 79 pairs of sectioned laserscan surfaces of adult female feet.

**Fig 4:**
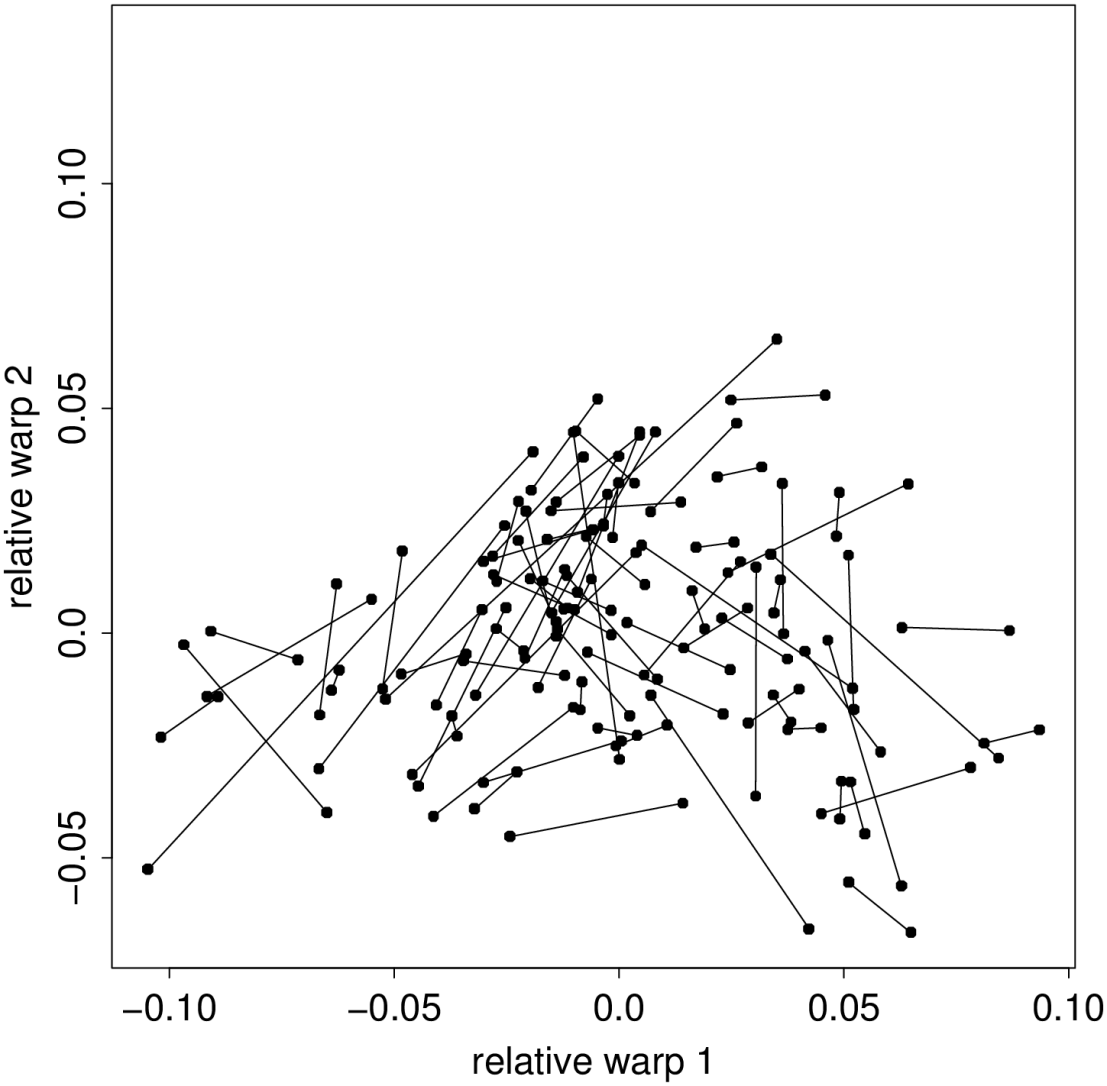
The first two relative warp scores for 79 pairs of insole outlines. Dashes link left and right foot of each subject.

**Fig 5:**
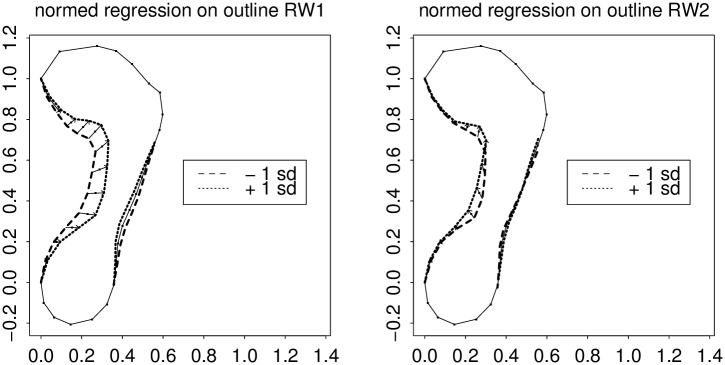
Geometry of these relative warps. (left) RW1, which looks like the sum of arch index and arch angle effects. (right) RW2, which looks like a geometric difference of the same two patterns. For explanation of the multiple lines on this figure, see the text. In this and subsequent figures, size has been normalized by fixing the locations of the ends of the long diameter of the form as shown. The length divided out would be a proxy shoe size measure except that it intentionally omits reference to the toes.

The coordinates resulting from the sliding algorithm, Fig 3, were submitted to one standard data manipulation of geometric morphometric (GMM) analysis (see, e.g., Chapter 4 of [32]). Geometric morphometrics carefully weaves information from geometry together with information from biology in order to more clearly highlight patterns from a class that the viewing eye (even the trained anthropologist’s eye) can see only dimly: the endlessly informative patterning of biological shape and shape change. Its procedures incorporate a standard strategy for the extraction of relative warps with respect to Procrustes distance (principal components of shape: [32], Sec. 4.4). The purposes of a principal component analysis are threefold: the reduction of dimensionality of a high-dimensional data set in order to simplify its description; the interpretation of the main dimensions of the underlying data distribution in biological terms; and the production of an ordination that represents the specimens of a sample as points on paper or in a simulated three-dimensional plot so that the investigator can discern clusters, trends, or outliers. Our application here emphasizes the first two of these purposes: the simplification of the 36-gons into just two dimensions, and the identification of those dimensions with simple functions (sum and difference) of the classic Clarke and Chippaux arch parameters. The third purpose is conveyed only in Fig 4, which shows that the left and right foot of a single subject resemble one another enough that these parameters can be considered pertinent to the person, not the specific foot. The restriction of the count of meaningful components to just two was per the stepdown rules of Bookstein. ([33], Sec. 5.2.3.4) Although the computation of these relative warps was itself wholly standard, Figs 5 through 9 here render them with both ends of the long diameter fixed (the so-called Bookstein coordinate representation). The visual impression of size standardization then corresponds to an anatomical length measurement rather than the more technical Centroid Size scaling built into the Procrustes toolkit.

This data resource differs from that of Domjanic [1] in a few details. Most obviously, the present paper excludes the outline information from the toes. Their length, in particular, is not a factor in the computation of the Procrustes size measure that is divided out in the course of standardizations such as that in Fig 3. The visualization of the relative warps has changed from a juxtaposition of filled outlines to a direct construction as simultaneous displacement of all the outline’s semilandmarks at the same time. We explicitly measured the two classical insole-relevant quantities mentioned above to see if the principal components of the footprint outline resembled them to any extent. Because they do, we can quantify that relationship by another standard tool, the technique of *shape regression* (regression of each element in a vector of scape coordinates against the same endogenous or exogenous scalar predictor). We did not anticipate the extent to which the conjecture that they were nearly equivalent as information resources would actually prove true.

#### Ethics statement

The study was reviewed and approved by the Ethics Committee of the University of Zagreb’s Faculty of Textile Technology before the study began. After receiving an explanation of the research and measurement protocols, each subject signed a consent form likewise approved by the Ethics Committee of the University of Zagreb’s Faculty of Textile Technology. Linking of physical measurements to questionnaire responses is by code number, never by name.

## Results

The general features of the Procrustes analysis of the semilandmarks, Fig 3, are familiar from other examples of the technique (compare, e.g., Fig. 4.34 of [32]). Points thrown into a coarse alignment by the slipping algorithm nevertheless are free to vary substantially in the direction perpendicular to the approximate local tangent line. This variability is concentrated in the region of the arch and posterior to the ball of the footprint. The relative warp analysis of these coordinates results in a series of empirical eigenvectors explaining, in order, 0.249, 0.109, 0.076, 0.076, …, units of Procrustes variance, which are 37%, 16%, 11%, 11%, … of the total shape variability in the data. (Shape variability here is coded as summed squared distance from the sample mean in the plot just displayed.) The rule for interpreting series of principal components finds that the ratio between the second and the third of these eigenvalues, 109/76 ∼ 1.43, exceeds the 50th percentile of such ratios for samples of size 100 (an approximation here taking account of the nonindependence of left and right feet of the same subject)—see the chart on page 324 of Bookstein [33]—but the ratio of the third and fourth certainly does not, and so it is appropriate to interpret up to two dimensions of the data set here.

The scatter of the first two eigenvector scores (in keeping with the standard methodology, see [32], Fig. 4.13, or [33], Fig. 6.7 or 7.13) is shown in Fig 4. Together these two dimensions cover 53% of the shape variation observed in these 158 outlines. The paired observations for left and right foot of the 79 subjects have been connected with line segments. The average squared length of these line segments is much less than it would be on an assumption of independent foot shapes: they average 58% of the sum of the variances of the two dimensions, versus the 200% they should average on an independence hypothesis—thereby confirming that shoes are best marketed in mirror-image pairs.

Fig 5, another standard plot of the GMM toolkit (see [32], Fig. 4.23, or [33], Fig. 7.23) diagrams the patterns of joint semilandmark displacement for each of these two dominant relative warps once an open-toe shoe size (long diameter of the form omitting the toes) has been normalized by fixing the locations of the ends of the long diameter of the form, as shown. (These are the so-called two-point shape coordinates or Bookstein coordinates of these points; see [34], Chapter 5.) Displayed are predicted locations for two sub-arcs of the outline (the interesting ones) for two specific values of a predictor that is just the relative warp score itself: a value one standard deviation below its average, and a value one standard deviation above its average. At left we see the pattern for relative warp 1, which, in this orientation, moves the arch outline and the posterior ball border inward or outward at the same time, and by nearly the same geometric extent. On the lateral side of the footprint is a similar effect of much lower amplitude.

At right is the same analysis for the second relative warp. Corresponding to its lower eigenvalue, it shifts points to a lesser net extent. More importantly, it represents a contrast between the arch narrowing and ball shortening that we saw as the action of RW1. When RW2 widens the arch, it shortens the ball of the foot, whereas RW1 would have lengthened it. Reading the same contrast in reverse, we could as well say that when RW2 narrows the arch, it also lengthens the ball, whereas RW1 would have shortened it. This sum-and-difference formulation is a typical interpretation of principal components extracted from highly correlated character suites: see, in general, [35].

**Fig 6:**
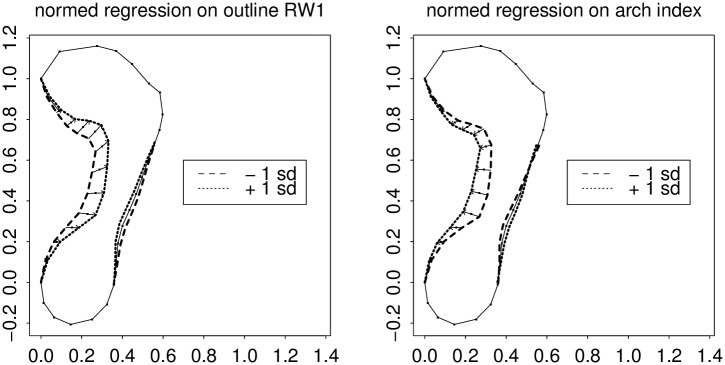
Relative warp 1 versus Chippaux’s index. Left, regression profile of semilandmark locations on the RW1 score; right, on the Chippaux-Šmiřák arch index as implemented in Fig 2. The Chippaux index shows a comparable effect at its locus of measurement, but falls off inefficiently toward the ball of the foot. The word “normed” in the title of this figure means that the regression coefficient is not in the usual units of the predictor variable per se (here, the RW score on the left, or the index score on the right) but instead takes on units of the predictor’s standard deviation. The arch index in the figure title is the ratio of the length of the lower straight-line segment length in Fig 2 to the length of the upper segment.

**Fig 7:**
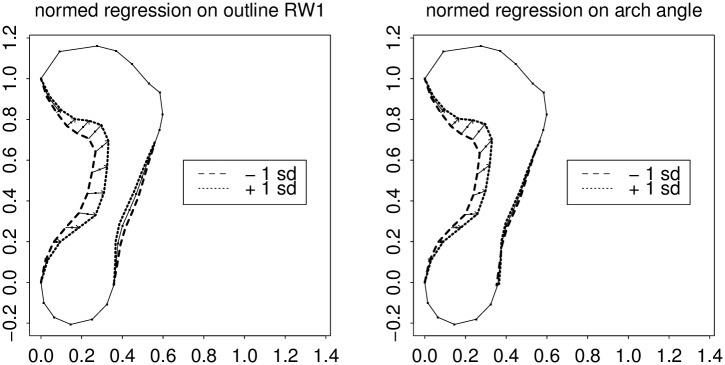
The same as Fig 6 for the comparison with arch angle. Clarke’s measure shows the same pattern as RW1 in the vicinity of the corner of the anteromedial arc, but falls off inefficiently toward the posterior arch.

The first relative warp, by itself, incorporates most of the effect of this pair (37% of Procrustes sum of squares versus 16%). Fig 6 copies the left panel of Fig 5, showing the action of this principal component (the pattern of regression coefficients when the outline shape coordinates are regressed individually upon this scalar), and compares it to the action of the arch index alone. As these predictors are in different units, the arch index effect has been normalized to the predictor’s own standard deviation just as in Fig 5. Arch index, as you see, has an effect as powerful as RW1’s at the locus where it is measured, but tapers off too quickly toward the front of the foot. Similarly, Fig 7 shows the regressions of the arch angle measure in this same scheme. This measure is as sensitive as RW1 right where the arch angle itself is measured, but tapers off posteriorly, along the arch, just as the action of the arch index tapered off towards the anterior.

**Fig 8:**
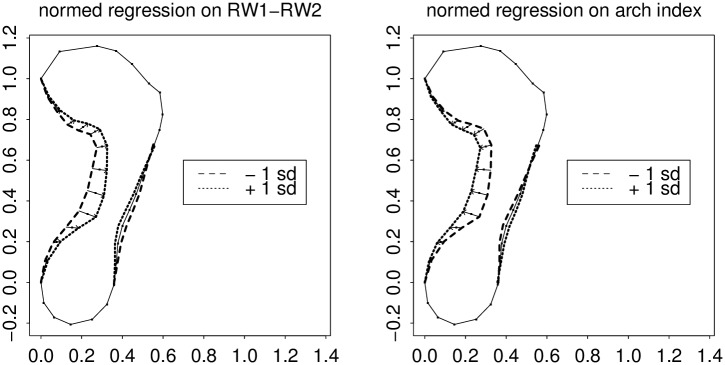
Regression of the semilandmark polygon on the difference of the first two RW scores, versus regressions on our version of the classic arch index. Left, predictions from the difference of RW’s; right, from the classic measure as implemented in Fig 2. The patterns seem synonymous.

**Fig 9:**
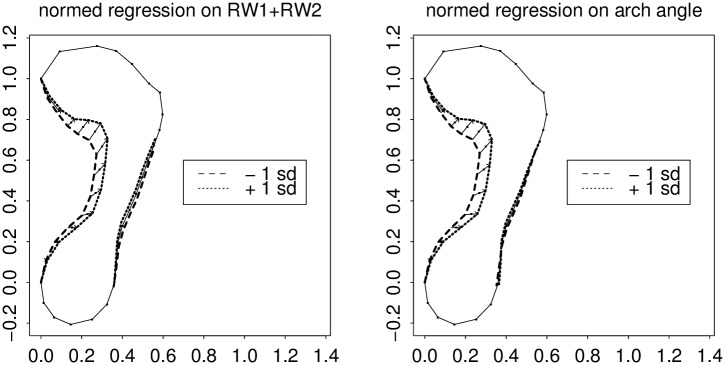
The same for Clarke arch angle (Fig 1) vis-à-vis the sum of the first two relative warp scores. Panels are as in Fig 8. Again the actions seem synonymous.

Because according to Fig 5 the action of RW2 is to contrast the effects on the anterior and posterior aspects of the outline segment here, we can see the same sum-and-difference construction in reverse, by substituting RW1–RW2 for the arch index measure and RW1+RW2 for the arch angle measure (Figs 8 and 9). (The arithmetic is executed in the domain of scores, not the domain of loadings.) The sum of the patterns of RW1 and RW2 closely matches the action of the arch angle measure. Analogously the difference of the same patterns, Fig 9, closely matches the action of the other main scalar index, the Chippaux-Šmiřák.

The corresponding plots of correlations of these sectional outlines by other measures of the women, such as body mass index, need for orthotics, or habitual use of high heels, are effectively null. Hence the two relative warps we have identified here should not be considered as consequences of interventions on these feet.

## Discussion

### Reassurance about conventional measurements

The role of geometric morphometrics as we have seen it in this example—to sharpen conventional measurement schemes by expansion into a spatial distribution—is typical of its applications all over applied anthropology. Earlier experts in amateur anthropometry found individual measurements that supported clinically useful comparisons; the role of the GMM tools is to understand the structure of those candidate indexes themselves. We saw in Fig 5 that our first two relative warps proved to be the difference and the sum of one particular pair of standard scalar indices, the Chippaux-Šmiřák arch ratio and the arch angle. The two measures are correlated −0.53 with each other, but each correlates about ±0.8 with our first relative warp—as we have seen, the value of that first relative warp, treated as a score for each of our 158 footprint outlines, is nearly equivalent in its information content to a linear combination of the two indexes taken together (R ∼ 0.94).

**Fig 10:**
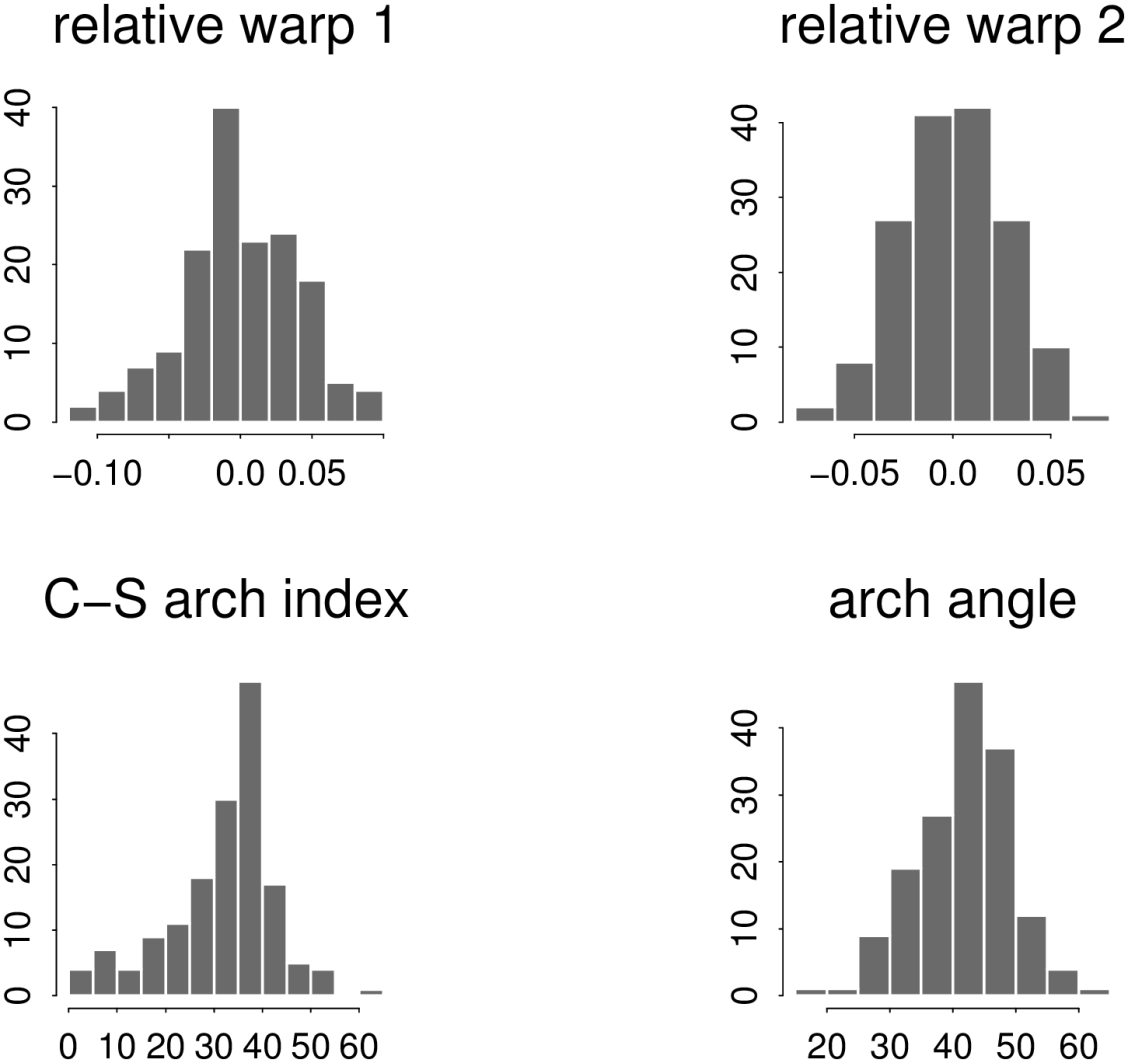
Dominance of the relative warp analyses over the classic indexes. This may owe to maldistribution of the standard measurements in comparison to the relative warps of the same outlines.

While the conventional indexes are not thereby shown to be unreasonable, nevertheless one particular formal property of principal component solutions, their role as optimal predictors, recommends them whenever they are computationally feasible. A possible explanation for this preference within the present data set is set out in Fig 10: the distribution patterns (each one over all 158 footprints) of the four quantities we are considering here. The top row shows our two relative warp scores; the bottom row, the pair of matching conventional indices (left, Chippaux-Šmiřák ratio; right, arch angle). While both of the relative warp distributions look approximately Gaussian, that for the Chippaux-Šmiřák index is obviously not: it is skewed to the left and much too sharply peaked. That would correspond to its clinical purpose, which is the detection of specific anomalies. Relative warp 2 looks particularly Gaussian in this representation. (In a context of detection, a bimodal distribution may be preferable to one with just a single mode. See, for instance, [36] for a contrasting application to the explicit diagnosis of *pes planus*.)

When GMM was introduced into anthropometry around the turn of the present century, by the efforts of teams in Vienna and elsewhere, one of the principal arguments accounting for its success was the demonstration that it obviated the need to continually guess at more and more conventional quantifications (indexes, proportions, angles) as the number of salient anthropometric points and curves continued to rise. (See the discussion in [32], Sec. 4.5.3.) The demonstration in the present manuscript constitutes a first step toward a similar possibility for the applied anthropology of clothing. To the extent that the two relative warps of these outlines together span the same information as a small system of conventional indicators (namely, the arch index and the arch angle), there is no further need for either of those indexes separately. The profession is free thereby to concentrate on the actual clinical matter at hand, which is the ordination of clinical subjects along a suitable number of dimensions of continuous variation bearing implications for the appropriate markets or therapies, in this context, prostheses. The design of index formulas was always an art, not a craft; one important consequence of the advent of GMM is the possibility of circumventing further needs for that high art.

The results here bear substantial implications for the marketing of orthotics. The first two relative warps, in particular, can serve as a template against which to simultaneously shift all the points of the margin at which an orthotic is designed to rise (to convey support) at the same time. The descriptive system here combines the effects of arch width variability and arch angle variability, while accommodating their moderate intercorrelation.

But beyond custom-fitted products, these findings bear implications for the broader market of prefabricated inserts. Within a given product line, typically shoes come in a two-parameter system, size (length) by width (perhaps AAAA to EEEE, perhaps narrow, medium, wide). Today’s insoles, however, seem not to be width-coded. If they were not custom-fitted by a professional from the details of the individual footprint, they were purchased from a store or a website that codes them by a single integer only, their overall size. Our results strongly suggest that there may be a rational design compromise midway between these two extremes: a second dimension of form (our first dimension of shape) that modifies the average insole of a particular size for feet whose arch-ball combinations are thinner than normal, thicker than normal, or within the normal range. A second dimension of shape, explaining roughly half as much variation as the first, represents the contrast between the two factors of that arch-ball combination rather than their sum.

In the same way that the manufacture of shoes turned to a two-dimensional system of prefabricated sizes (length by width, in equal increments), the manufacture of insoles could be converted from the present one-dimensional system (just an integer size, to be modified by the pedestrian’s own scissors) to a three-parameter system that could still be marketed in prefabricated packets hanging from a compact display rack, but that would much more closely track the actual population variation of geometries by which the insole actually varies—according to the figures here, that is by a combination of foot size, arch angle, and arch ratio.

### Fulfilling the vision of biostereometrics

Just as dress design patterns modify the geometry of a fixed template for a range of physical sizes and extents of obesity, this three-dimensional insole scheme would incorporate the best contemporary quantifications of arch form, adapting the earlier insights of Clarke and Chippaux to the present context of three-dimensional surface-based anthropometry. We will thereby have taken a major step forward toward bridging the concerns of enterprises like Project CAESAR [37], based as they are on actual population ranges of a fixed roster of a-priori parameters, with the more flexible approach to human morphological variation appropriate for data from dense meshes, [38] the data type that characterizes most 21st-century research into human clothing manufacturing patterns (e.g., [39]). This was the original goal of biostereometrics as it coalesced around the new surface-scanning technologies in the 1970’s (see [40]). The promise of that new information resource proved inseparable from the generation of the corresponding biometric technologies for averaging and correlating the information thus acquired.

In this paper we demonstrate one contemporary bridge of that sort, the match of relative warps of outline shape to the best guesses at relevant dimensions of form-variation as imagined and diagrammed more than half a century ago. We commend this thrust as a fertile domain for advancing the technological use of information about the variation of human forms in general. Today’s statistics are quite capable of confirming, rather than replacing, the judgments of the founding generation of anthropometricians.

## Supporting Information

S1 Text Semilandmark coordinates and indices. This text file is a listing of of 158 × 36 ordered pairs, the Cartesian coordinates of the semilandmarks described in the Methods section above, along with two decimal values, the classic arch index (Fig 2) and the arch angle (Fig 1), for the same 158 cases. The format of the file is 158 lines of 72 Cartesian coordinates and then two decimal index values per line. Arch angle is in units of degrees.
(TXT)
